# Computational Intelligence-Based Model for Mortality Rate Prediction in COVID-19 Patients

**DOI:** 10.3390/ijerph18126429

**Published:** 2021-06-14

**Authors:** Irfan Ullah Khan, Nida Aslam, Malak Aljabri, Sumayh S. Aljameel, Mariam Moataz Aly Kamaleldin, Fatima M. Alshamrani, Sara Mhd. Bachar Chrouf

**Affiliations:** Department of Computer Science, College of Computer Science and Information Technology, Imam Abdulrahman Bin Faisal University, Dammam 31441, Saudi Arabia; iurab@iau.edu.sa (I.U.K.); msaljabri@iau.edu.sa (M.A.); saljameel@iau.edu.sa (S.S.A.); 2170007806@iau.edu.sa (M.M.A.K.); 2170006481@iau.edu.sa (F.M.A.); 2170007790@iau.edu.sa (S.M.B.C.)

**Keywords:** COVID-19, mortality rate, prediction, machine learning, deep learning

## Abstract

The COVID-19 outbreak is currently one of the biggest challenges facing countries around the world. Millions of people have lost their lives due to COVID-19. Therefore, the accurate early detection and identification of severe COVID-19 cases can reduce the mortality rate and the likelihood of further complications. Machine Learning (ML) and Deep Learning (DL) models have been shown to be effective in the detection and diagnosis of several diseases, including COVID-19. This study used ML algorithms, such as Decision Tree (DT), Logistic Regression (LR), Random Forest (RF), Extreme Gradient Boosting (XGBoost), and K-Nearest Neighbor (KNN) and DL model (containing six layers with ReLU and output layer with sigmoid activation), to predict the mortality rate in COVID-19 cases. Models were trained using confirmed COVID-19 patients from 146 countries. Comparative analysis was performed among ML and DL models using a reduced feature set. The best results were achieved using the proposed DL model, with an accuracy of 0.97. Experimental results reveal the significance of the proposed model over the baseline study in the literature with the reduced feature set.

## 1. Introduction

COVID-19 is a highly contagious disease caused by a novel coronavirus. It was first detected around the end of 2019 in Wuhan, China. In January 2020, the virus was announced as a global issue by the World Health Organization (WHO). The virus affects people in diverse ways. However, it is known that elderly people and those with chronic diseases are more likely to experience severe illness [1]. This has put enormous strain and pressure on governments and healthcare organizations, as well as on their limited resources. According to statistics provided by the WHO on 8 February 2021, more than 105 million confirmed cases have been reported globally, with nearly 2 million confirmed deaths caused by the virus [2].

The ongoing COVID-19 pandemic is causing a massive number of infections worldwide due to severe acute respiratory syndrome (SARS-CoV-2). Due to the exponential rise in the number of infections and mortality rates, monumental pressure has been placed on governments and public healthcare systems. It is thus essential to identify critical factors of mortality to optimize patient treatment strategies. Moreover, for COVID-19, it was found that hospital capacity is related to the mortality rate; the lower the hospital capacity, the higher the mortality rate [3]. Thus, it is important to prioritize patients with an elevated risk of mortality and to identify the features of COVID-19 patients that lead to mortality [4].

Artificial Intelligence (AI) offers a unique opportunity to improve the response to this widespread disease as it continues to evolve and spread. Specifically, ML and DL techniques can help in the rapid discovery of insights and show relationships across large populations of heterogeneous COVID-19 cases. These methods can help to automatically extract and identify the relationship among complex attributes. In this study, several ML and DL based models were developed to predict the mortality risk of COVID-19 cases using a worldwide clinical record of COVID-19 cases. The proposed models could help hospitals to assign priorities to patients with a potentially higher risk of mortality. The study compared the ML and DL models developed to predict the rate of mortality in COVID-19 cases using sociodemographic and clinical data.

This paper is organized as follows: Section 2 presents the background of the COVID-19 pandemic and the application of AI to control it. Section 3 presents a review of related studies. In Section 4, the material and methods are described. Section 4 describes the experimental set-up, results, and discussion. Finally, in the last section, the conclusion and recommendations for future research are discussed.

## 2. Background Section

The WHO created a framework that describes an infectious disease breakout in six distinct stages [5]. The first is the “Identification” stage, where a new contagious virus or bacteria has been found. The next stage, “Recognition”, involves pinpointing where the disease clusters are located around the world. Thirdly, “Initiation” is where sustained human transference is established. The following stage is “acceleration”. During this interval, case rates speed up, and in tandem, several mitigation methods are deployed. These deployed methods cause a levelling-off period and a subsequent reduction in the number of cases, which leads to the next stage, “deceleration”. As the rate of spread slows, the world is launched into the “preparation” stage to prepare for the subsequent pandemic wave.

AI has the potential to address challenges at all these stages. This section will provide a brief description of how AI can be used to identify, prevent, address, and recuperate from the coronavirus. For example, AI systems in China identified the emergence of a novel strain of pneumonia [6]. Moreover, Deep Learning (DL) can be used to develop new drugs to treat COVID-19. Several companies, such as DeepMind, are employing DL to find prototype treatments and vaccinations for COVID-19 (SARS-CoV-2) [7]. As COVID-19 is now considered to be a global pandemic, AI can be utilized by policymakers, healthcare organizations, and communities to help manage each phase of the disease.

AI can be employed to help identify, recognize, and fend off further transmission of the disease. Several algorithms capable of identifying any patterns or abnormalities are already used to identify and predict where the spread of COVID-19 will be highest. Furthermore, the use of AI-augmented image processing and recognition accelerates the diagnosis process. Moreover, artificial intelligence can assist in recognizing the chain of how the virus spreads and can carry out surveillance on a wide range of economic effects. In several instances, AI has been shown to infer epidemiological data faster than conventional health data surveying. Organizations such as the Organization for Economic Co-operation and Development (OECD) and Johns Hopkins University have developed interactive dashboards that monitor how the virus is spreading using data from the news regarding new cases of COVID-19, new recoveries, and new deaths. It is important to note that, in order to limit the spread of the coronavirus, there is a need to accelerate the diagnostic process. Inadequate testing for COVID-19 has hindered the identification of the disease in affected individuals. Alternatives that depend on radiology, such as CT scans and X-rays, have been shown to out-perform serology and viral tests (PCR and antigen tests).

As part of their disease prevention strategies, several countries track COVID-19 cases using population surveillance tools. For instance, South Korea traces COVID-19 cases by employing programs that use data referring to the geographical location of patients, data from surveillance cameras, and credit card records. China allocates everyone a colour-coded risk level, which indicates their individual contagion risk [8]. Machine learning models use records of travel, credit card payments, and communication to assign the appropriate colour and predict the location of the next disease outbreak. Furthermore, multiple countries use geolocation to identify people who have been in contact with known virus carriers, a process known as contact tracing. Additionally, autonomous systems, such as robots and drones, have been developed to respond to emergencies in hospitals.

AI systems can also be used to tackle this health catastrophe through the use of misinformation correction and to disseminate personalized information regarding suitable advice and treatment. To combat the threat of misinformation, search engines such as Google and social networks such as Facebook use algorithms that remove any false or misleading information from their platforms and provide users with personalized knowledge using AI. Moreover, chatbots and online assistants have been developed to answer people’s queries and to assist hospitals in triaging people based on the presence of symptoms. Furthermore, AI has been employed to find patients at an elevated risk of death. For example, the Medical Home Network implemented an AI-augmented system that uses the risk of respiratory difficulties to identify high-risk patients.

In summary, artificial intelligence can be used in the detection of COVID-19, either for early warning systems to detect anomalies or any digital “smoke signals” or for diagnosis where AI is employed to recognize patterns in medical imagery and symptom data. For the prevention phase of the COVID-19 crisis, AI can be employed to calculate the probability that a person will be infected, a concept known as prediction. AI can also be used for surveillance, monitoring and tracking the contagion in real-time, and information, personalized news and content moderation to fight misinformation. For the response stage, AI is employed in delivery, where, for example, drones are used to transport materials, and robots are utilized for high-exposure tasks at hospitals. AI can also be employed for service automation where chatbots and digital triaging assistants are deployed. Lastly, AI can be used in recovery to monitor and track economic recovery using Global Positioning System(GPS), satellite, and social media data.

## 3. Related Studies

The significance of ML and DL in many fields has motivated many researchers to use these techniques widely during the COVID-19 pandemic for automated diagnosis, prognosis, triaging and the creation of tools capable for predicting the mortality rate of severe COVID-19 cases. These models can help healthcare professionals monitor cases remotely in a resource-constrained system. Some recent studies concerning the mortality rate prediction of COVID-19 patients are discussed below.

### 3.1. Machine Learning Based Approach

Aljameel et al. [9] developed several machine learning models to predict the severity of symptoms in COVID-19 patients. The dataset consisted of 287 COVID-19 patients who were admitted to the King Fahad university hospital in Kingdom of Saudi Arabia (KSA). Several feature selection techniques and classification algorithms were compared, such as LR, RF, and XGBoost. RF produced the best results with an accuracy of 0.95 using 20 features.

Ko et al. [10] aimed to develop an Ensemble-based Deep Neural Network (EDRnet) using an ensemble learning method to predict in-hospital mortality using routine blood samples that are administered at the time of admission. The dataset consisted of 73 blood biomarkers from 467 COVID-19 patients. In total, 28 blood biomarkers, together with age and gender information, were selected using analysis of variance (ANOVA) and available data rate (ADR) feature selection techniques. An ensemble approach was adopted combining RF models with a DNN, which achieved 1 sensitivity, 0.91 specificity, and 0.92 accuracy. To increase the number of patient points, the authors also developed an online web application (BeatCOVID-19) to predict mortality by providing blood test results. An improvement to the research would be to update the model to train it on data collected from multiple countries and regions.

Similarly, authors in [11] applied ML techniques to clinical data of COVID-19 patients under treatment at the Mount Sinai Health System (MSHS) in New York City to predict mortality. The training set consisted of 3841 patients, of whom 313 died and 3528 are alive. The retrospective set consisted of 961 patients of whom 78 are deceased and 883 are alive. Lastly, the prospective set consisted of 249 patients, of whom 25 are deceased and 224 are alive. Recursive feature elimination was applied for feature selection. Furthermore, several ML models, such as Support Vector Machine (SVM), Random Forest (RF), Logistic Regression (LR), and Extreme Gradient Boosting (XGBoost), were applied and reached an Area Under Curve (AUC) value of 0.91. It was found that the patient’s age, minimum oxygen saturation throughout their medical treatment, and inpatient vs. outpatient status were the key features for prediction. The only limitation of this study is the limited features collected. The dataset only included features that are routinely collected during hospital treatment.

Likewise, the authors in [12] also used XGBoost and developed a COVID-19 Mortality Risk (CMR) tool for hospitalized COVID-19 cases. The dataset contains 3062 COVID-19 patients from six centers located at 33 hospitals in the USA and countries in southern Europe. A total of 2302 patients are alive and 760 are deceased. Patient demographics, clinical data, patient symptoms and comorbidities, and laboratory test results were acquired at the time of admission. The researchers trained a binary classification model, based on the XGBoost learning algorithm, to predict the outcome (1 for deceased and 0 for discharged). The model achieved an AUC of 0.901, an accuracy of 0.85, and a Negative Predictive Value (NPV) of 0.93. They found that elevated CRP, BUN, glucose, creatinine, platelet counts, and aspartate aminotransferase (AST) are highly indicative laboratory features, aside from age and low oxygen saturation. Conversely, the limitations of the study concern biases in terms of how limited hospital resources may have caused an increased rate of mortality.

Moreover, the authors in [13] used several ML techniques to identify five key features that assist in predicting COVID-19 patient mortality. The study used a dataset consisting of 1766 attributes of 370 COVID-19 cases. Missing data were imputed using K-Nearest Neighbor (KNN); however, MinMaxScalar was used for normalization. Several models, such as SVM, LR, XGBoost, RF, Decision Tree (DT) and Neural Network (NN), were used for classification. NN achieved an accuracy of 0.965, an F1 score of 0.969, and an AUC of 0.989. Lymphocytes, neutrophils, high-sensitivity C-reactive protein (he-CRP), lactate dehydrogenase (LDH), and age were identified as the most indicative biomarkers using NN and XGBoost with backward step-down feature selection techniques.

Comparatively, [14] analyzed data from 4098 COVID-19 patients who were admitted to the MSHS Hospital in New York. SHapley Additive exPlanations (SHAP) and Least Absolute Shrinkage and Selection Operator (LASSO) feature selection techniques were used to extract the most distinguishing features. Several ML models were compared, and it was found that XGBoost outperformed all other models in predicting in-hospital mortality and other crucial events at time periods of 3, 5, 7, and 10 days from the date of admission. It achieved the highest AUCs of 0.89 for predicting mortality at three days.

Furthermore, a similar study was conducted by Das et al. [15] in South Korea to predict the mortality of COVID-19 cases using ML. They used a public dataset published by the Korea Centers for Disease Control and Prevention containing 3524 COVID-19 patients from 20 January to 30 May 2020. Five ML models (LR, SVM, KNN, RF, and Gradient Boosting (GB)) were used. LR outperformed the other models, with an accuracy of 0.968 and AUC of 0.830. The limitation of the proposed tool was the unavailability of clinical information on COVID-19 patients. In addition, the authors did not use a hold-out set that had not been previously used for validation. Furthermore, it may introduce overfitting.

Similarly, the authors in [16] used the Korean data to develop ML models to predict COVID-19 patients’ prognosis. The dataset contains the clinical and demographic information of the patients in South Korea. It was created using a combination of the database of patients with a confirmed positive COVID-19 test. Of the 10,237 patients, 7772 recovered, 228 died, and 2237 were still being treated. Several models were used, such as LASSO, SVM (Linear), SVM (Radial Basis Function (RBF)), RF, and KNN. The best-performing models were LASSO and SVM (Linear). LASSO achieved an AUC of 0.963 and a balanced accuracy of 0.911. L1-norm feature selection techniques were used with SVM in order to select the most important features. However, SVM (Linear) achieved an AUC of 0.962 and a balanced accuracy of 0.919. The study found a strong association between mortality risk and attributes such as age, gender, disability, the existence of symptoms, diabetes mellitus, and asthma.

Comparatively, researchers in [17] analyzed the cases of COVID-19 in Madrid using ML and survival analysis techniques to predict mortality. The dataset was acquired from the HER system of HM hospitals and contained 29 variables from admission and clinical data from 2307 patients. Multiple data analysis methods were used, such as survival data analysis, LR, Bayesian Network (BN), DT, RF, and biclustering. LR achieved the best outcome with an AUC of 0.89, a sensitivity of 0.81, and a specificity of 0.81. The study confirms that older people have an elevated risk of dying from COVID-19. Some of the highly significant attributes were oxygen saturation, (emergency) ER simplified diagnostic, and ER department. Additionally, decision rules for predicting the mortality risk of COVID-19 patients can be deduced from DTs and help clinicians triage COVID-19 patients. They could identify different patient groups by using unsupervised learning, which allows the global analysis of drugs distributed to patient populations. However, the study is limited to data from a certain population that was collected under complex health situations.

Moreover, [18] aimed to help triage patients by developing ML models to predict COVID-19 mortality of ventilated ICU patients. The dataset used was a combination of the Medical Information Mart for Intensive Care (MIMIC) dataset, which contained data of ~60,000 ICU admissions, and a dataset formatted in the same way as MIMIC from a community hospital, which contained data from 114 patients. XGBoost was used with a learning rate of 0.1 and 1000 tree for tree ensembles to limit the computation, and the optimization of hyperparameter was done using cross-validated grid search. Results showed using the MIMIC dataset for training and testing the XGBoost obtained for mortality prediction of ventilated ICU patients with an AUC of 0.82, 0.81, 0.77, 0.75 for hour windows of, 12, 24, 48, and 72 h, respectively. The community hospital dataset for testing the mortality prediction the AUC values were 0.91, 0.90, 0.86, 0.87 for hour windows of 12, 24, 48, and 72 h, respectively. However, the study is limited to ICU patients and does not consider patients of other care settings. It also only focuses on laboratory data and does not incorporate care team actions. Lastly, the study requires prospective validation to determine mortality prediction performance.

Additionally, [19] used RF to predict mortality risk for COVID-19 patients with time-series clinical data. The dataset contains 567 COVID-19 patients. Gini importance criteria were used with RF in order to select the most important features. The model scored an accuracy of 0.655 and AUC of 0.855. However, validation is required for the model, and its generalizability is limited given that variables such as treatment and intervention are not included. In addition, the patients’ level of care was not taken into consideration due to the small sample size.

Correspondingly, researchers in [20] developed a predictive model for COVID-19 patients in Nigeria. The dataset used consisted of 302 responses from an online survey based on ten basic factors that cause mortality for COVID-19 patients. Several models were used, such as multilayer perceptron with 20 layers, DT, NB, and decision rule. The outcome of the study showed that the highest accuracy was achieved by implementing multilayer perceptron with a value of 0.85.

Furthermore, Tezza et al. [21] analyzed clinical data from 341 COVID-19 patients collected from hospitals in Italy. Age, lab measures (creatinine, platelets, AST, hemoglobin, and lymphocytes), and vital signs (quick Sequential Organ Failure Assessment (SOFA) and oxygen saturation) had the highest impact on the models’ outcome and performance. Recursive Partition Tree (RPART), SVM, Gradient Boosting Machine (GBM), and RF were used. ML models were built on the extracted data to predict in-hospital mortality rates. The best results with Receiver Operation Characteristics (ROC) value of 0.84 were achieved with the RF model.

Similarly, Ferreira et al. [22] analyzed patient data from the Directorate-General of Health of Portugal by applying different models such as LR, NB, DT, and Artificial Neural Networks (ANN). These models aim to predict COVID-19 patients’ outcomes, i.e., recover or deceased. The best results achieved were extracted using all the available comorbidities, age, and symptoms information along with the oversampling sampling technique and DT algorithms. A sensitivity value of 0.95, accuracy of 0.90, and specificity of 0.86 were achieved. These models were used as a part of a clinical decision system that could help authorized users take action towards COVID-19 patients.

Comparably, [23] analyzed data from 1955 COVID-19 patients from the HM Hospitals Group in different areas of Spain. Several measures were analyzed using the models: age, gender, neutrophil-to-platelet-ratio (NPR), neutrophil-to-lymphocyte-ratio (NLR), C-reactive-protein level, oxygen saturation, and the rate of changes of both hemogram ratios (VNLR (neutrophil-to-lymphocyteratio) and VNPR(neutrophil-to-platelet-ratio)) during week 1 from the date of admission. An LR ML model was built on the data with 10-fold cross-validation to predict the mortality rates of COVID-19 patients. LR showed the best performance when analyzing VNLR with AUC value of 0.891. The proposed model can be used for the early detection and prediction of in-hospital mortality risk in Spain. Furthermore, a study [24] used COVID-19 dataset from 146 countries to predict the mortality rate. Several machine learning models like SVM, ANN, RF, DT, LR, and KNN were used. The study results showed that ANN model has achieved the highest accuracy of 0.8998 using 57 features.

Table 1 contains a summary of related studies using the ML model to predict COVID-19 patients’ mortality.

### 3.2. Deep Learning (DL) Based Approach

The study [25] aimed to predict ICU admission and in-hospital mortality of COVID-19 patients using DL and risk-score system. The dataset consisted of clinical information from 1108 patients, of whom 837 were general admissions, and 271 were admitted to the ICU. Of the 837 general admissions (772 were discharged and 65 died), there were 271 ICU admissions (86 were still in the ICU, 108 were discharged, and 77 died). All patients were admitted to Stony Brook University Hospital in New York. Feature raking was performed using Boruta, which uses Random Forest to rank features. The identified top predictors were then fed to a DL model that consists of five fully connected dense layers. For the hidden layers, the ReLU activation function was used, while the sigmoid activation function was used for the output layer. To create the risk-score model, the Generalized Additive Model was used to graph and illustrate the probability of ICU admission and mortality for each independent clinical variable. For mortality prediction, the proposed model achieved an AUC of 0.844 and an accuracy of 0.853. It was found that the significant features for mortality are procalcitonin, age, LDH, CRP, cardiac troponin, and SpO2. However, comorbidities were not a significant predictor of ICU admission and mortality.

Furthermore, Dhamodharavadhani et al. [26] investigated Statistical Neural Network (SNN) models, such as Generalized Regression Neural Network (GRNN), Radial Basis Function Neural Network (RBFNN), and Probabilistic Neural Network (PNN) for COVID-19 mortality prediction in India. They used the Novel Corona Virus 2019 Dataset, which contains data collected from patients listed in India’s death cases. The dataset is further divided into two datasets: D1, containing a time series of death cases, and D2, containing two attributes, an independent attribute “confirmed case” and a predictive attribute “death case” of COVID-19. Each of the three SNN models is trained once with D1, and later with D2, and performance is then evaluated using RMSE and the correlation coefficient. To improve accuracy, a hybrid model was constructed by combining the Non-linear Autoregressive Neural Network (NAR-NN) with SNN models. The results show that RBFNN and PNN had the best performance in both datasets. PNN with an RMSE:8.88, R:0.99 in D1, and RMSE:7.89, R:0.99 in D2. RBFNN had RMSE:8.52 R:0.99 in D1 and RMSE:9.50 R:0.99 in D2. The SNN models had better performance when D1 was used compared to D2. Thus, the hybrid models constructed are used with D1. It was also found that the D1 RBFNN model performed best, while in D2 PNN performed best. The first limitation of this study is the under-reporting of COVID-19 cases in India. In addition, the SNN models do not consider the demographic and geographical factors related to the spread of COVID-19. Lastly, the hybrid model SNN-NAR-NN is suited for short-term mortality prediction.

Additionally, Zhu et al. [27] proposed a triaging model for COVID-19 patients using a DL model to derive a risk stratification score system and identify the top clinical variable predictor. The dataset contains 78 clinical variables of 181 cases with COVID-19 infection from a Chinese hospital. To measure its performance, the prediction model was compared with the COVID-19 severity score, pneumonia severity index (PSI), and CURB-65 score. The top five features and risk score had, respectively, an AUC value of 0.968 and 0.954. The model outperformed the COVID-19 severity score with an AUC = 0.756, PSI with an AUC = 0.838, and a CURB-65 score with an AUC = 0.671. For the mortality prediction rates, the risk stratification score system (0–5) scored 0%, 0%, 6.7%, 18.2%, 67.7%, and 83.3%, respectively. The study is limited to a single hospital, and only clinical variables were considered; including longitudinal clinical data could provide a better insight. Furthermore, mortality may depend on the resources and patient load of the hospital, which could vary between different countries.

Similarly, Li et al. [28] used two datasets from two Spanish hospitals (HM, H120) containing clinical data from 3780 and 2307 COVID-19 confirmed cases and built a temporal sequence of the clinical information of each patient. A Recurrent Neural Network (RNN) with an attention mechanism was used. The proposed RNN is composed of four models: an embedding module, recurrent module, classifier module, and optional attention modules, which use daily records of static temporal data to produce a daily prediction. The model was compared with SVM and RF. Several experiments were conducted in a different setting, including two competitive baselines, different dataset splits, hyper-parameter tuning, feature selection mechanism (entropy, Gini index, information gain, and chi-square), exhaustive evaluation method, and adequate model selection. Performance was assessed using a global metric by taking the average of outcomes of all days. For the HM dataset, the best RNN had no penalty in F1 and had a sensitivity score of 0.84 for all folds, and for the attention mechanism, 56.59% of patients had peak days. For the H120 dataset, the ensemble-SEN model had a sensitivity score of 0.80 for all folds, and for the attention, 66.36% of patients had peak days. In terms of performance using day-by-day predictions from the outcome date, the models had better results than baselines. However, the data were limited to two hospitals. More data would thus give a better understanding of the best procedures and medication for patients.

Table 2 contains a summary of related studies using Deep Learning models. 

All the studies mentioned above show the significance of ML and DL models in predicting mortality risk in COVID-19 cases. These prediction models can provide an integral tool for the remote monitoring and triaging of COVID-19 patients, especially in underdeveloped countries where resources are limited. These models can also help in the early identification of at-risk patients. Due to the exponential number of COVID-19 infected patients and numerous emergent strains of COVID-19, there is still a need to develop an intelligent automated system for predicting the mortality of COVID-19 patients. Therefore, the current study attempts to propose a model to predict the mortality of COVID-19 patients by applying ML and DL models on a large dataset. This study aimed to produce a better-performing model to help health professionals predict at-risk patients at an early stage.

## 4. Materials and Methods

**Figure 1:**
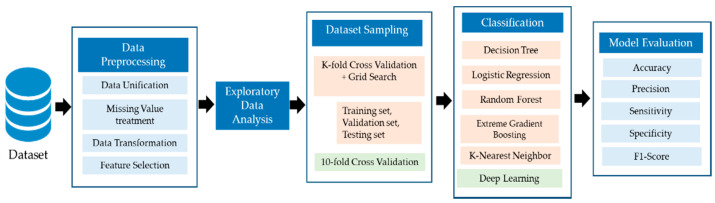
Framework of the proposed study.

The study was implemented using the Python programming language and Jupyter IDE. Sklearn version 0.23.2 library was used for conventional ML models. For the DL model, the TensorFlow version 2.4.1 was used. The model was trained on Intel core i7 and 16 GB RAM. The hash function was used to convert object type columns into integer type to enable them to be used and analyzed by the ML and DL algorithm. The dataset was then split using K-fold cross-validation with the value of K = 10. For the cross-validation, the dataset was initially divided into two sets, i.e., training and testing. The training set was then further divided into two folds, i.e., training and validation folds. The validation fold was used for parameter tuning. Finally, six different algorithms using ML and DL techniques were trained and tested along with parameter optimization to achieve the best outcome. Figure 1 represents the block diagram for the proposed study. The section below also includes a description of all the steps used in the proposed methodology.

### 4.1. Data Preprocessing

Data preprocessing is a crucial step in the development of ML and DL models. Several preprocessing techniques were applied, such as data unification, transformation, and missing value imputation. The study used an open-source dataset introduced in [29]. Initially the dataset consisted of 2,676,311 COVD-19 patients containing several outcomes, such as alive, transferred, migrated, death, dead, discharged, critical, recovered, severe, and stable. Some of the categories in the outcome have the same meaning, such as, death, dead, died, and deceased. Therefore, data unification was performed by replacing these with deceased. Some patient records with missing outcomes were removed from the dataset. The aim of the study was to predict the mortality rate of COVID-19 patients; consequently, the outcome was converted into two labels, i.e., deceased and survived. The final dataset contained 103,888 records, with 97,941 records from surviving patients and 5947 for deceased patients.

The Length of Stay (LOS) attribute was derived by subtracting the date of death/discharge from the date of admission. Some of the records in the dataset have either date of admission or date of death/discharge, or both these dates, missing. Therefore, for those records, the LOS was imputed with 0. After calculating the LOS attribute, three attributes were removed: date of admission, date of symptoms, and date of death/discharge.

**Figure 2:**
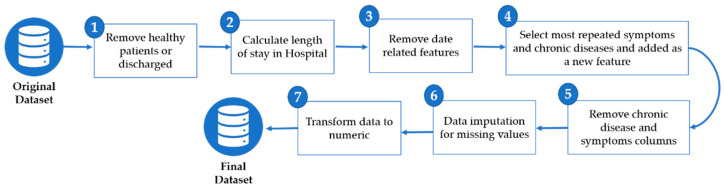
Data preprocessing steps.

There are various symptoms and chronic disease attributes in the dataset. Some of the symptoms and chronic diseases occur very infrequently and may only account for less than 10% of the entire dataset. Therefore, these attributes were removed. All symptoms and chronic disease attributes were selected where the percentage of occurrence is equal to or greater than 10%. Subsequently, one hot encoding technique was applied for each selected symptom and chronic disease. Furthermore, it was found that the gender column had a substantial number of missing values, which were replaced with the “Unknown” category. Lastly, the data were transformed to numeric values by encoding the categorical data. Figure 2 summarizes the pre-processing steps.

**Figure 3:**
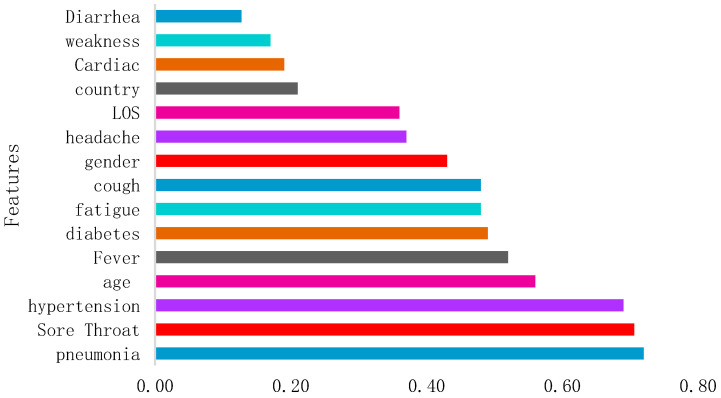
Selected features correlation in the dataset.

The selected attributes in the dataset were age, gender, country, LOS, fever, fatigue, weakness, pneumonia, cough, diarrhea, sore throat, headache, hypertension, diabetes, and cardiac disease. Thus, the dataset contains 15 features in total out of 32 from the original dataset. Figure 3 represents the selected feature correlation in the dataset. Figure 3 represents the correlation of the features with the outcome.

### 4.2. Exploratory Data Analysis

Researchers collected and curated heterogynous data on an individual level from different national, provincial, and municipal health reports, along with data from online reports. The geocoded data included travel history and symptoms, along with critical dates, such as onset date, admission data, and confirmation data. Initially, the dataset included over 2,676,311 records from over 76 countries, including records for males and females of all ages. After preprocessing, records from 103,888 patients from 45 different countries were left, with the highest number of patients from India (98,632). The second highest number of patients was from the Philippines (4493). However, there were less than 200 patients from each of the remaining countries. The analysis of the dataset is shown in Table 3. It contains the feature type, datatype, and unique values of the dataset features for each feature.

The dataset contains 97,165 patients with unknown gender. However, 3761 patients were female and 2960 male. The minimum length of stay (LOS) for the patient is 0 and the maximum LOS is 43 days. The most frequent LOS in the dataset is 7. Similarly, the minimum age of the patient is 0 years, the maximum age is 101, and the median age is 50. Fever, sore throat, and pneumonia are the most commonly occurring symptoms in the dataset. Similarly, in terms of chronic diseases, hypertension is the most common.

### 4.3. Predictive Algorithms

In this study, several DL and ML classifiers, such as deep neural network (DNN), DT, LR, RF, XGBoost, and KNN, were used to predict the mortality rate of COVID-19 patients. A grid search was used to optimize ML classifier parameters. A brief description of classifiers is given below.

- Decision Tree (DT)

Decision trees (DT) is a supervised ML algorithm that is used for regression and classification problems. A DT model uses a tree of decisions to reach a conclusion about a target variable, which is usually represented in the leaves of the tree. Trees can be classified as either classification trees, where the leaf contains discrete values, or as regression trees, where the leaf contains continuous values. Constructing a tree requires a split at the root into subsets, and the split is repeated on each subset. This process is recursive in nature; thus, the algorithm is known as a greedy algorithm, and it stops when no value is added to the prediction or when all nodes have the same value as the target value. The DT model is easy to understand and performs feature selection implicitly.

There are two main advantages to using DT:

○ The explanatory models provide a useful description about a phenomenon, unlike more complex models such as neural networks, where extracting knowledge is complex.
○ The non-parametric model assumes that the distribution of data is not defined by a finite set of parameters. During the learning process, the tree can grow depending on the problem’s complexity.

However, it can produce a complex tree that does not generalize well and causes overfitting.

- Logistic Regression (LR)

Logistic Regression (LR) is a supervised ML algorithm that is widely used for classification problems, mostly for binary class values. The algorithm name is based on its core function, the logistic or sigmoid function. The sigmoid function is used to map predicted values to probability scores between 0 and 1.

There are two main operations for this algorithm. The first is a linear operation from which the predictor or independent features are used to obtain multiple linear regression. The second applies the sigmoid function to obtain the probability score to a given class.

Sigmoid function:

(1) $$P\left(class|x_{1},..,x_{N}\right)=\frac{1}{1+e^{-\left(w_{0}+\sum_{k-1}^{N}w_{k}x_{k}\right)}}$$

where $w_{k}$ is the parameter of the model, and $x_{k}$ are the features for an object, and *N* is the total number of attributes.

The LR algorithm is fast in classifying new records and performs well for many simple datasets that are linearly separable. However, the algorithm may overfit in the case of high dimensional datasets, although this can be avoided when using regularization techniques [30]. In the Logistic Regression model used in this study, the following hyperparameters were tuned:

- penalty: to specify the penalization norm;
- random_state: for shuffling the data;
- max_iter: the maximum number of iterations for the algorithm to converge;
- tol: this refers to the tolerance for the stopping criteria [31].

Table 4 below presents the parameter values set used in LR.

- Random Forest (RF)

Random Forest (RF) is a supervised ensemble ML algorithm. It builds multiple decision trees known as a forest based on multiple samples of the training data. This technique is an extension of bagging, which is known to reduce the high variance characteristic of decision trees. It differs from bagging in that data is added to the tree at each split point in the data, and only a fixed set of features are considered [32]. The algorithm is used to solve classification and regression problems. Trees are generated using stochastic recursive partitioning, which randomizes each tree using two methods. To start with, randomizing for each tree is done by basing it on a smaller bootstrap sample from a larger training sample. The algorithm chooses a smaller set of samples from n data with a replacement so that every sample is either not chosen or chosen multiple times. Every bootstrap sample has 37% left out, which is known as out-of-bag and used as an error estimation. Second, at each node, it randomizes the tree by stochastically selecting a subset of attributes. Therefore, during training, the trees are partitioned so that the tree nodes are associated with objects that have similar predictable values. Following this, training samples are assigned to leaf nodes and the construction of the tree ends. In our RF algorithm the following hyperparameters were tuned:

- n_estimators: the number of generated trees;
- max_depth: the maximum levels in each tree;
- min_samples_split: the minimum number of node data before it splits;
- min_samples_leaf: the minimum amount of data that can populate the leaf node [33].

Table 5 below presents the parameter values set used in the RF.

- Extreme Gradient Boosting (XGBoost)

Extreme Gradient Boosting, also known as XGBoost, is a widely used ML algorithm. An implementation of gradient boosted decision, XGBoost is designed to provide both performance and speed. Moreover, because XGBoost uses parallelism, it is known for its ability to learn quickly and scale appropriately to the problem. In simple terms, XGBoost uses an ensemble method that implements the principle of boosting weak learners, and the gradient descent algorithm to enhance the model’s performance [34,35]. For example, when using the algorithm for regression, the weak learners are a regression tree. For each tree, it assigns the input data to leaf nodes that hold continuous values. The algorithm reduces a regularized function combined with the model complexity penalty term, which is the function of regression trees and a convex loss function that depends on the difference between the target and predicted outputs. Training is carried out in an iterative process by adding new trees that predict the errors of previous trees, which are later combined with the previous trees to make a final decision or result.

For a dataset (X,Y):

(2) $$F_{k}\left(X\right)=F_{k-1}\left(X\right)+\alpha_{k}h_{k}\left(X,r_{k-1}\right)$$

(3) $$\arg\underset{\alpha }{\min}=\sum_{i=1}^{k}L\left(Y_{i},F_{i-1}\left(X_{i}\right)+\alpha h_{i}\left(X_{i},r_{i-1}\right)\right)$$

whereL(Y,F(X)) a differntiable loss functio

Here, $r_{i}$ and $\alpha_{i}$ are regularization parameters, and $h_{i}$ is a trained function that predicts residuals $r_{i}$.

XGBoost has many different parameters that alter its performance. The two parameters altered in this experiment are objective and random_state. Objective defines the loss function meant to be minimized, while random_state refers to the random number seed. Other parameters were left to their default values [36]. Table 6 below represents the different parameters and the values chosen in this experiment.

- K-Nearest Neighbor (KNN)

K-Nearest Neighbors, also known as KNN, is a lazy supervised learner. A lazy model refers to the fact that the model stores the whole dataset given to it and classifies new data based on similarity measures (usually distance). KNN assumes that similar things will exist close to each other in the same area. It can be used for classification and regression problems. KNN calculates the distance between the points based on the similarity measure and labels the unknown data depending on the values of the closest neighbours in proximity to it. KNN has only one hyperparameter that affects its performance, which is K. K is the number of closest neighbours that can be considered after deciding the label of unknown data sample [37]. For numerical data, to find the nearest neighbour to calculate the distance between the new sample and every training sample, the distance is calculated using either Euclidean, Makowski, or Manhattan distances. For categorical data, the Hamming distance is used. The distance formulas are as follows:

Euclidean:

(4) $$\sqrt{\sum_{k=1}^{N}{\left(x_{k}-y_{k}\right)}^{2}}\;$$

Makowski:

(5) $${\left[\overset{N}{\underset{k=1}{\sum }}{\left(\left|x_{k}-y_{k}\right|\right)}^{q}\right]}^{1/q}$$

Manhattan:

(6) $$\overset{N}{\underset{k=1}{\sum }}\left|x_{k}-y_{k}\right|$$

where $x_{k}$ is the test sample attribute value while $y_{k}$ is the training sample attribute value, and *N* is the total number of attributes.

Hamming:

(7) $$d_{ij}=l+m$$

where *l* is the number of variables with one category for *i* object, and *m* is the number of variables with the other category for *j* object. KNN can produce good results for many tasks, such as categorizing texts, recognizing patterns, detecting objects, and ranking models. However, there are some disadvantages, such as memory consumption, as it stores the whole dataset, and it is prone to perform badly when there is noise in the data.

For this experiment, the value of *k* was equal to 5.

- Deep Learning Model

Deep Learning models, also known as Deep Neural Network (DNN), are a type of neural network (NNs) that contain three main layers: input, output, and hidden layers.

○ Input layer: in this layer, the values of inputs *x_n_* enter the neural network. For each input, the weight *w_n_* is applied, before their summation is sent to the hidden layer in which the activation function *ƒ* is used.
○ Hidden layer: in this layer, the activation function *ƒ* uses the summation from the input layer including *a* bias *b* and sends it to all nodes in the next hidden layer, which eventually produces the output variable *y_n_*.

(8) $$y_{n}=f\left(b+\sum w_{n}x_{n}\right)$$

○ Output layer: in this layer, the output value is determined with the lowest error rate possible, and backpropagation is applied to reduce the error rate by using the gradient descent algorithm to adjust new weights *Wn*.

(9) $$W_{n}=w_{n}-a\left(\frac{\partial Error}{\partial w_{n}}\right)$$

The network may have many hidden layers, each of which depends on the output of the previous layer. The equation below demonstrates the output of layer *n* in the network, where *f*, *b*, and *w* are activation function, bias, and weights, respectively, and *y_n_*_−1_ is the output of the previous layer.

*y_n_* = *f* (*w_n_* · *y*_*n*−1_ + *b_n_*) (10)

DLs are usually Feed Forward Networks (FFNNs) in which the data goes only in one direction from the input layer to the output layer. DNN usually consists of many hidden layers depending on the problem complexity and show efficient results in pattern recognition and classification.

The proposed DL model architecture consists of six fully connected dense layers with 19 variables as inputs, where each layer consists of 512, 512 (dropout_layer), 128, 128 (dropout_layer), 64, 64 (dropout_layer), and 1 (output_layer) neurons, respectively. The three dropout layers with a 20% dropout rate were added to allow the model to learn more robust features. Moreover, rectified linear units (ReLU) were chosen as activation functions. The output layer used a sigmoid function because binary classification was used to classify patients into deceased and survived.

ReLU activation function:

(11) R(x)=max(0,x)

where if *x* is negative the output is 0, and thus, the node will not be activated; few nodes are active, making the neural network sparse and efficient.

Sigmoid activation function:

(12) $$\sigma \left(x\right)=\frac{1}{1+e^{-x}}$$

where the range of values for the output is from [0,1]. The output is equal to 0.5 when *x* is equal to 0. The output gets closer to 1 when *x* is a huge positive number and gets closer to 0 when *x* is a huge negative number.

The Adam optimization algorithm with learning rate 0.001 was used for model optimization, while “binary_crossentropy” was employed to calculate the loss of the model. The accuracy metrics were used to evaluate the accuracy of the model. For training the model, the epochs were set to 50 and batch size to 512, along with the additional setting of callbacks, where the monitor was set to “Validation accuracy”, save best only to “True” and mode to “max”. The Kfold cross validation with n_splits were set to 10 to optimize model performance.

### 4.4. Evaluation Measures

To evaluate the proposed models, a wide range of different performance measures were used. These include the following:

Accuracy is the ratio of correctly predicted COVID-19 cases to the total number of COVID-19 cases. Accuracy is mathematically shown in Equation (13).

(13) $$Accuracy=\frac{TrueSurvivedCOVID-19patients+TruedeceasedCOVID-19patients}{TotalnumberofCOVID-19patients}$$

*Precision* is the ratio of the correctly predicted records for the surviving COVID-19 patients’ class to the total predicted as survived COVID-19 patients. High precision corresponds to a low false-positive rate [38]. This can be seen mathematically in Equation (14).

(14) $$Precision=\frac{TruepredictedSurvivedCOVID-19patients}{predictedsurvivedCOVID-19patients}$$

*Sensitivity* is the ratio of correctly predicted patient records for the survived COVID-19 patients’ class to the total survived patients in the dataset [38]. This is mathematically described as shown in Equation (15):

(15) $$Sensitivity=\frac{TruepredictedSurvivedCOVID-19patients}{Totalnumberofsurvivedpatientsinthedataset}$$

*Specificity* is the ratio of correctly predicted deceased COVID-19 patients to all the deceased patients in the dataset [39]. This is mathematically described, as shown in Equation (16):

(16) $$Specificity=\frac{TruepredicteddeceasedCOVID-19patients}{Totalnumberofdeceasedpatientsinthedataset}$$

*F*1 − *Score* is the weighted average of recall and precision, taking both false negatives and false positives into account [38]. Mathematically this is represented as shown in Equation in (17):

(17) $$F1-Score=\frac{2*\left(Sensitivity\times Precision\right)}{Sensitivity+Precision}$$

## 5. Results and Discussion

In severe cases, COVID-19 can lead to multiple organ damage, acute respiratory distress, and death. Therefore, it is essential to identify patients at an elevated risk of mortality early on and to support them by providing them with appropriate treatment and care. Moreover, the shortage of resources and staff poses a major challenge to healthcare systems and governments. The identification of high-risk patients can help solve this challenge by allowing these institutions to better plan and optimize the use of their limited technical and human resources. In this study, six different classifiers were used to predict mortality in patients. These models include DT, LR, RF, XGBoost, KNN, and the DL model.

**Figure 4:**
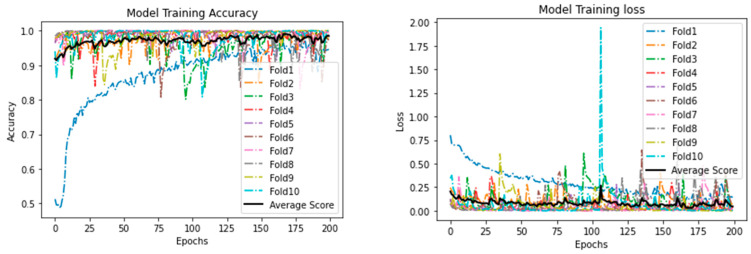
Proposed Deep Learning model: training accuracy and loss.

**Figure 5:**
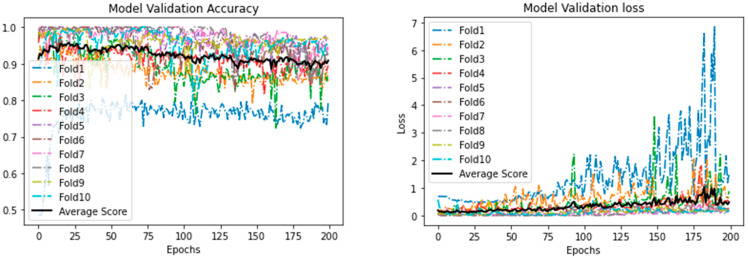
Proposed Deep Learning model: validation accuracy and loss.

Table 7 shows the results obtained from the different models using the 15 selected features. As shown, all models achieved very comparable results. It can be seen that the DL model outperformed ML models with respect to all the evaluation measures, as it achieved an accuracy of 0.970, precision and specificity of 1.000, sensitivity of 0.970, and F1−Score of 0.985. The best performing ML models are RF and XGBoost. Both achieved an accuracy of 0.946. Moreover, both the models had high precision (0.998), sensitivity (0.947), and F1-score (0.972). It is important to note, however, that XGBoost outperformed RF with regard to specificity, with XGBoost achieving a specificity of 0.810 and RF achieving a specificity of 0.807. The worst-performing model was KNN, accuracy, precision, specificity, and F1-score of which was lower than for the other models. Figure 4 shows the training accuracy and loss, and Figure 5 represents validation accuracy and loss.

In medical applications, the false-negative rate should be minimized as much as possible, as people who test positive for a particular disease should take the necessary precautions and receive the care and treatment they need. Especially in the case of this pandemic, it is imperative that people with COVID-19 are aware of their condition so that they refrain from spreading their infection to others and take all precautionary measures required. Therefore, one of the most important measures in this research is sensitivity. A high sensitivity corresponds to a low false-negative rate. Among all the reviewed literature, this research resulted in the highest sensitivity of 0.970.

Moreover, although false positives are not as significant as false negatives in medicine, it is still important to minimize the false positive rate. False positives have the associated hassle of staying at home for 14 days, unable to carry out responsibilities. Of the reviewed literature, this research also provides the highest precision of 1.000.

Because both the sensitivity and precision achieved in the proposed research is higher than the studies mentioned in the literature, the F1 score obtained is also the highest, namely, 0.985. The F1 score is one of the most important measures in the current study because it takes into consideration the subtleties of class imbalances and/or the varying cost of false positives and false negatives. Furthermore, [24] uses the same dataset as that used in the current study. While they achieved a reported accuracy of 0.9375, this research surpassed that accuracy, with an acquired value of 0.970.

This study aimed to develop DL and ML models using sociodemographic and brief clinical (symptoms and patient comorbidities) attributes. The proposed models can better contribute to the fight against the pandemic and provide medical institutions and governments with a way to identify high-risk patients so that they can provide them with the support they need and save lives. The proposed model outperformed the baseline study as shown in the Table 8.

Despite all these advantages, there is always a room for improvement. The proposed model needs to be investigated on other datasets. Similarly, there is a need to include other features that were identified as significant in the literature review. Moreover, it will be important to explore the impact of these features on the models’ performance.

## 6. Conclusions and Recommendation

Announced as a global pandemic on 2 February 2020, COVID-19 can lead to multiple organ damage, acute respiratory distress, and death. It has presented governments and healthcare institutions with the never-ending nightmare of attempting to reduce the mortality rate of the disease with limited technical and human resources. This study aims to help fight COVID-19 by developing a model capable of discerning high-risk patients early on to enable institutions to optimize their human and technical resources, thus ensuring that all patients receive the care and support they need from the outset.

The main contribution of the proposed study is:

- Comparative analysis of the DL and ML models in predicting the mortality rate of COVID-19 patients;
- The study proposed a model to achieve better results with reduced number of features as compared to the baseline study;
- In general, the proposed DL model outperformed the baseline study in terms of all performance evaluation measures. The model can be an effective tool for the early prediction of at-risk COVID-19 patients.

In this study, one DL and five ML models were developed using sociodemographic and brief clinical data to predict mortality. Models included DL, DT, LR, RF, XGBoost, and KNN. The study showed that the DL model outperformed the other five classifiers, with an accuracy of 0.97, a precision of 1, a sensitivity of 0.97 a specificity of 1, and an F1-score of 0.985. It is hoped that this study will help fight the pandemic. Despite achieving significant results, the proposed model needs to be investigated with other datasets. Similarly, there is a need to include the lab findings to further explore the significant features.

The proposed model can be used as a decision support system to identify patients at a high risk of mortality. Furthermore, models can help with the remote triaging of the COVID-19 patients, thus reducing the burden placed on hospitals and the risk of contamination.

## Figures and Tables

**Table 1:** Summary of related studies using machine learning models.

Reference	Technique	Dataset Size	Feature Selection	Results
[9]	RF	287 COVID-19 patients	Extra tree classifiers	ACC:0.95 AUC: 0.99
[10]	Ensemble based on RF and DNN.	467 COVID-19 patients	ANOVA, ADR	ACC: 0.92 SPE: 0.91 SEN: 1
[11]	SVM, RF, LR, XGBoost	3841 COVID-19 patients	Recursive feature elimination	AUC: 0.91
[12]	XGBoost	3062 COVID-19 patients	-	AUC: 0.901 ACC: 0.85 NPV: 0.93
[13]	NN	370 COVID-19 patients	backward step-down	ACC: 0.965 F1 score: 0.969 AUC: 0.989
[14]	XGBoost	4098 COVID-19 patients	SHAP, LASSO	AUC: 0.89
[15]	LR	3524 COVID-19 patients	-	ACC: 0.968 AUC: 0.830
[16]	SVM(Linear)	10,237 COVID-19 patients	L1-norm	AUC: 0.963 ACC: 0.911
[17]	LR	2307 COVID-19 patients	-	AUC: 0.89 SEN: 0.81 SPE: 0.81
[18]	XGBoost	~60,000 patients	-	AUC: 0.91
[19]	RF	567 COVID-19 patients	Gini importance criteria	ACC: 0.655 AUC: 0.855
[20]	MLP	302 responses from an online survey	-	ACC: 0.85
[21]	RF	341 COVID-19 patients	-	ROC: 0.84
[22]	DT	-	-	SEN: 0.95 ACC: 0.90 SPE: 0.86
[23]	LR	1955 COVID-19 patients	-	AUC: 0.891
[24]	ANN	3,073,82l COVID-19 patients	-	ACC: 0.8998

**Table 2:** Summary of the related studies using Deep Learning models.

Reference	Technique	Dataset Size	Feature Selection	Results
[25]	DL with 5 condensed layers	1108 COVID-19 patients	Boruta	AUC: 0.844 ACC: 0.853
[26]	RBFNN, PNN	-	-	RMSE:7.89 R:0.99
[27]	DL	181 COVID-19 patients	-	risk score AUC: 0.968 severity score AUC: 0.756 PSI AUC: 0.838 CURB-65 score AUC: 0.67
[28]	RNN	3780 and 2307 COVID-19 confirmed cases from 2 datasets	Entropy, information gain, Gini index, chi-square	SEN: 0.84

**Table 3:** Description of the dataset.

Feature Type	Feature Name	Datatype	Values (Unique)
Demographic	Age	Numeric	101
	Sex	Categorical	3
	Country	Categorical	76
Hospital Attribute	LOS	Numeric	34
Symptoms	Fatigue	Categorical	2
	Fever	Categorical	2
	Weakness	Categorical	2
	Pneumonia	Categorical	2
	Cough	Categorical	2
	Diarrhoea	Categorical	2
	Sore Throat	Categorical	2
	Headache	Categorical	2
Chronic Disease	Hypertension	Categorical	2
	Diabetes	Categorical	2
	Cardiac	Categorical	2
Target	Outcome	Categorical	2

**Table 4:** Optimized parameter values for the Logistic Regression model.

Parameter	Value
Penalty	l2
Random_state	777
Max_iter	10,000
Tol	10

**Table 5:** Optimized parameter values for the Random Forest model.

Parameter	Value
n_estimators	100
max_depth	15
min_samples_split	5
min_samples_leaf	1

**Table 6:** Optimized parameter values for Extreme Gradient Boosting model.

Parameter	Value
Objective	binary: logistic
Random_state	42

**Table 7:** Performance comparison of the proposed models for mortality rate prediction of COVID-19 patients.

Classifier	Accuracy	Precision	Sensitivity	Specificity	F1-Score
Decision Tree	0.945	0.998	0.947	0.799	0.972
Logistic Regression	0.945	0.998	0.946	0.777	0.972
Random Forest	0.946	0.998	0.947	0.807	0.972
XGBoost	0.946	0.998	0.947	0.810	0.972
K-Nearest Neighbors	0.944	0.997	0.947	0.699	0.971
DNN	0.970	1.000	0.970	1.000	0.985

**Table 8:** Comparison of proposed model with the baseline study.

Reference	Year	Techniques	Features Used	Accuracy
[24]	2021	NN	57 features	0.8998
Proposed study	2021	DNN	15 features	0.970

## Data Availability

The study used an open-source dataset accessible from the weblink https://github.com/beoutbreakprepared/nCoV2019 (accessed on 18 April 2021).
